# Induction of antimicrobial, antioxidant metabolites production by co-cultivation of two red-sea-sponge-associated *Aspergillus* sp. CO2 and *Bacillus* sp. COBZ21

**DOI:** 10.1186/s12896-024-00830-z

**Published:** 2024-01-17

**Authors:** Ahmed A. Hamed, Mosad A. Ghareeb, Ayda K. Kelany, Mohamed Abdelraof, Hoda A. Kabary, Nariman R. Soliman, Mohamed E. Elawady

**Affiliations:** 1 Microbial Chemistry Department, National Research Centre, 12622 Dokki, Cairo, Egypt; 2 Medicinal Chemistry Department, Theodor Bilharz Research Institute, Kornaish El-Nile, 12411 Warrak El-Hadar, Imbaba, Giza, (P.O. 30), Egypt; 3 Department of Genomic Medicine, Cairo University, Giza, Egypt; 4 Department Agricultural Microbiology, National Research Center, 33 El Buhouth St., Dokki, 12622 Giza, Egypt; 5 Dairy Science Department, National Research Center, Dokki, Cairo Egypt; 6 Microbial Biotechnology Department, Biotechnology Research Institute National Research Centre, Cairo, Egypt

**Keywords:** Coculture, Fungi, Bacteria, Antimicrobial, Antioxidant, Antibiofilm

## Abstract

The growing spread of infectious diseases has become a potential global health threat to human beings. According to WHO reports, in this study, we investigated the impact of co-cultivating the isolated endophytic fungus *Aspergillus* sp. CO2 and *Bacillus* sp. COBZ21 as a method to stimulate the production of natural bioactive substances. (GC/MS)-based metabolomics profiling of two sponge-associated microbes, *Aspergillus* sp. CO2 and *Bacillus* sp. COBZ21, revealed that the co-culture of these two isolates induced the accumulation of metabolites that were not traced in their axenic cultures. By detection of different activities of extracts of *Bacillus* sp. COBZ21 and *Aspergillus sp.* CO2 and coculture between *Bacillus* sp. COBZ21 *and Aspergillus sp.* CO2. It was noted that the coculture strategy was the reason for a notable increase in some different activities, such as the antimicrobial activity, which showed potent activity against *Escherichia coli* ATCC 25,922, *Staphylococcus aureus* NRRLB-767, and *Candida albicans* ATCC 10,231. The antibiofilm activity showed significant biofilm inhibitory activity toward *Bacillus subtilis* ATCC 6633, *Pseudomonas aeruginosa* ATCC 10,145, *and Staph aureus* NRRLB-767, with activity up to 53.66, *71*.17, *and* 47.89*%*, while it showed low activity against *E. coli* ATCC 25,922, while the antioxidant activity based on the DPPH assay showed maximum activity (75.25%). GC-MS investigations revealed the presence of variable chemical constituents belonging to different chemical categories, which reflected their chemical diversity. The main components are (+-) cis-Deethylburnamine (2.66%), Bis(3,6,9,12-tetraoxapentaethylene) crowno-N,N,N’,N’-tetra methylpphanediamine (2.48%), and 11-phenyl-2,4,6,8-tetra(2-thienyl)-11-aza-5,13-dithiaeteracyclo[7.3.0.1(2,8)0.0(3,7)] trideca-3,6-diene-10,12,13-trione (3.13%), respectively, for *Bacillus* sp. axenic culture, *Aspergillus* sp. CO2, *Aspergillus* sp. CO2, and *Bacillus* sp. COBZ21 coculture. By studying the ADME-related physicochemical properties of coculture extract, the compound showed log *P*_o/w_ values above 5 (8.82). The solubility of the substance was moderate. In order to provide a comprehensive definition of medicinal chemistry and leadlikness, it is important to note that the latter did not meet the criteria outlined in the rule of three (RO3). The toxicity prediction of the coculture extract was performed using the ProTox II web server, which showed that the selected compound has no pronounced toxicity.

**Supplementary Information:**

The online version contains supplementary material available at 10.1186/s12896-024-00830-z.

## Background

Antimicrobial resistance is one of the significant challenges of the 21st century. The appearance of new infectious and multidrug-resistant microorganisms is emerging as a new threat to human health and global stability [1]. The imperative to identify novel medications with unique modes of action derived from natural sources is indisputable as a means to address the emergence of antimicrobial resistance and ensure their safety and efficacy [2]. Multi Drug Resistance (MDR) pathogens represent one of the most critical global health threats for both human and feed stocks due to their increased resistance to the known antibiotic categories, which makes the disease caused by one of the MDR groups inevitably lead to death.

In the ecological system, there are two distinct forms of microbial competition that might take place: interference competition and scramble competition. Interactions can occur either intra-specifically or inter-specifically. Interference competition occurs when a certain microbe actively restricts access to nutrients for another microorganism. The phenomenon of scramble competition occurs when a certain microbe depletes available nutrients prior to another microorganism. Indeed, the level of competition escalates in situations where there is limited availability of nutrients. Microbial co-culture exhibits considerable promise in facilitating the identification of continuous production of secondary metabolites, a feat that can be accomplished within controlled laboratory settings. Recent discoveries have highlighted the significance of mixed fermentation or microbial co-culture, as well as microbial transformation, genome mining, and unculturable bacteria, as crucial sources of new antibiotics [3].

Previous studies have extensively examined co-cultures, resulting in findings such as enhanced production of known metabolites or the discovery of new substances. The microbial co-culture technique, which involves replicating conditions seen in nature, has been shown to enhance the efficacy of antibiotics in crude extracts, augment the production of established secondary metabolites, generate analogs of known metabolites, and activate bioactive component pathways that were previously dormant. The fungal co-culture strategy has proven to be a successful method for activating static biosynthetic gene clusters in fungal strains, resulting in the production of hitherto undiscovered secondary metabolites. This strategy typically encompasses three distinct approaches, namely fungal-fungal, fungal-bacterial, and fungal-host co-cultures [4, 5].

In previous studies, the co-cultivation of two marine microorganisms, namely the fungus *Emericella* sp. and the actinomycete *Salinispora arenicola*, resulted in a significant enhancement of the expression of the emericellamide biosynthetic gene cluster 100-fold [6]. The co-cultivation of the marine-derived fungus *Libertella* sp. with the marine bacteria CNJ-328.8 resulted in the induction of the creation of novel cytotoxic diterpenes, namely libertellenones A-D. A further illustration entailed the synthesis of a novel antibiotic, pestalone, using the co-cultivation of the marine fungus *Pestalotia* sp. alongside an unidentified unicellular marine bacterium, strain CNJ-328 [7].

Therefore, the current study aimed to use a coculture strategy between fungal and bacterial isolates to increase the activity of different activities such as antimicrobial, antioxidant, and antibiofilm.

## Materials and methods

### Collection of sponge sample

**Fig. 1:**
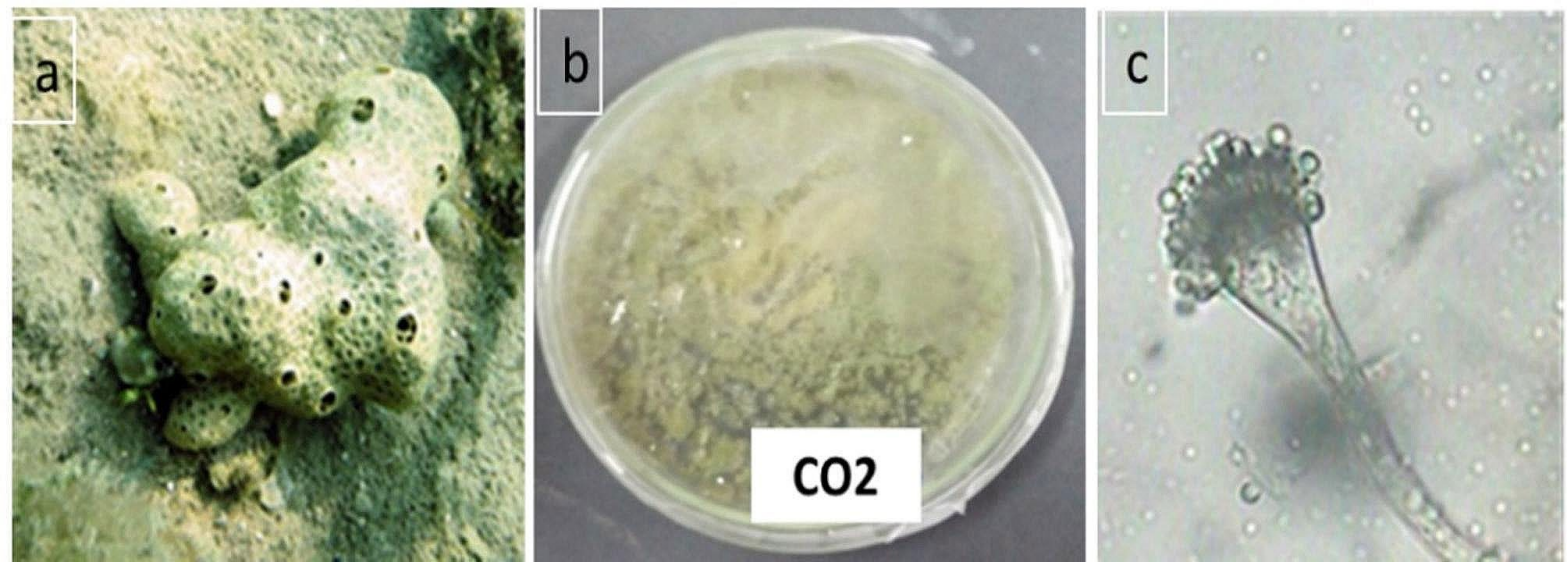
**(a)** Marine sponge morphology **(b)** Colony morphology of the fungus on PDA **(c)** Microscopic examination of the isolated fungus

The marine sponge *Corella cyathophora* was collected using SCUBA equipment from Hurghada at N 26°59’42.87”, E 33°54’4.02” at 10 m depth in the Hurghada region. Red Sea, Egypt. The collected sponge was brought into the laboratory, coded, photographed (Fig. 1), and kept in a cold place (5 °C) until the isolation of fungal and bacterium strains.

### Isolation of sponge-associated fungus and bacteria

The isolation of fungus and bacterium associated with the sponge commenced with the implementation of a surface sterilization procedure. This involved subjecting the sponge to a 1-minute treatment with 75% ethanol, followed by a 3-minute immersion in a solution of 0.5% sodium hypochlorite. Subsequently, the sponge was rinsed three times with sterilized, distilled H_2_O, and let to air dry under aseptic conditions. Following this, the desiccated segments of the sponge were crushed using a mortar. Subsequently, 1 gram of the powdery material was suspended in 9 milliliters of sterilized distilled water and vigorously mixed using a vortex mixer until a uniform mixture was achieved. Serial dilutions were performed up to the third order, and subsequently, 1 ml of the second and third dilutions were aseptically put onto Petri dishes containing rose Bengal agar. The Petri dishes were then incubated at a temperature of 30 °C for a duration of 5 days. Following the completion of the incubation period, the fungal colonies that had grown were then placed onto potato dextrose agar plates and subjected to incubation under the identical circumstances as before [8]. While, bacterial isolate was is incubated at a temperature of 37 °C for a duration of 3 days on nutrient agar plat.

### Morphological identification

The most potent fungal isolate was selected based on antimicrobial screening (data not showed). The phenotypic characterization of the most potent fungal isolate was conducted by cultivating the fungus on potato dextrose agar plates for 10 days. The analysis encompassed the inspection of colony morphology, including shape, color, and medium pigmentation, as well as microscopic evaluation of the produced spores and mycelium.

### Genetic identification of the isolated fungus and bacteria

The process of molecularly identifying fungal (CO2) and bacterial isolates involved the extraction of genomic DNA using the Qiagen DNeasy Mini Kit, following the guidelines provided by its designer [9]. The amplification reactions for fungal 18Sr RNA were performed using two primers: ITS1 (5TCCGTAGGTGAACCTGCG-3) and ITS4 (5-TCCTCCGCTTATTGATATGC3), and for bacterial 16Sr RNA, two primers: 27 F (5AGAGTTTGATCCTGGCTCAG-3) and 1492R (5’GGTTACCTTGTTACGACTT’3). The PCR products were submitted to two commercial sequencing services, namely SolGent and Macrogen, located in South Korea. The sequences that were acquired were subjected to analysis using the BLASTN algorithm in order to examine their similarity and homology with the relevant target gene sequences present in the NCBI database. The construction of the phylogenetic tree was performed using the maximum-likelihood (ML) method, facilitated by the software MEGAX [10].

### Coculturing

In the co-fermentation experiment, a 10 mL aliquot of a 3-day-old culture of *Bacillus* sp. COBZ21 was aseptically put into each of ten 2 L Erlenmeyer flasks. These flasks had previously been filled with 500 mL of ISP2 medium and inoculated with a 10 mL aliquot of a 5-day-old culture of *Aspergillus* sp. CO2.

### Extraction of bioactive secondary metabolites

Following the fermentation process of axenic cultures and co-culture, a filtration step was carried out. Subsequently, the resulting supernatant was subjected to extraction using ethyl acetate (1.5 L), resulting in the formation of the ethyl acetate soluble fraction (800 mg) [11].

### Antimicrobial activity by plate assay method

The antimicrobial efficacy of the examined extracts was assessed against various microbial strains, including Gram-negative bacteria (*Escherichia coli* ATCC 25,922, *Klebsiella pneumoniae, and Salmonella typhi*), Gram-positive bacteria (*Staphylococcus aureus* NRRLB-767, Methicillin-resistant *Staphylococcus aureus* (MRSA), yeast (*Candida albicans* ATCC 10,231*)*, and fungi (*Aspergillus niger* ATCC 10,231) [12, 13]. The experiment was conducted using 96-well flat polystyrene plates. A volume of 10 µl of test extracts, with a final concentration of 250 µg/ml, was mixed into 80 µl of lysogeny broth (LB broth). Subsequently, 10 µl of bacterial culture suspension in the logarithmic growth phase was added. The plates were then subjected to overnight incubation at a temperature of 37 °C. Following incubation, the observed antibacterial action of the examined extracts was manifested by clearance in the wells. Conversely, extracts that did not have any effect on the bacteria resulted in the growth media appearing opaque in the wells. The control group consisted of the pathogen without any kind of treatment. The measurement of absorbance was conducted around 20 h following the initiation of the experiment at wavelength 600 using a Spectrostar Nano Microplate Reader developed by BMG LABTECH GmbH, located in Allmendgrun, Germany.

### Biofilm inhibitory activity

The biofilm inhibitory activity of the extracts obtained was assessed using a microtiter plate assay (MTP) conducted in 96 well-flat bottom polystyrene titer plates against four clinical microorganisms (*Pseudomonas aeruginosa* ATCC 10,145, *Staphylococcus aureus* NRRLB-767, *Escherichia coli* ATCC 25,922, and *Bacillus subtilis* ATCC 6633) [14]. In this experiment, each well was filled with 180 µL of LB broth, which consisted of tryptone (10 g), yeast extract (5 g), and NaCl (10 g/L). Subsequently, 10 µL of an overnight pathogenic bacterial culture was inoculated into each well. Following this, 10 µL of the desired samples were added to the experimental wells, while a blank control was used as a reference. The entire setup was then incubated at a temperature of 37 °C for a duration of 24 h. Following the incubation period, the contents within the wells were extracted and subsequently subjected to a washing process using 200 µL of phosphate buffer saline (PBS) at a pH of 7.2. This procedure was carried out in order to eliminate any bacteria that were not attached to the surface. The sessile bacteria were immobilized using a 2%sodium acetate solution and subsequently stained using a 0.1% crystal violet dye. The excessive discoloration was eliminated through a process of washing with deionized water and thereafter being lowed to dry. Additionally, the dried plates underwent a washing process with 95% ethanol. Subsequently, the optical density (OD) was measured at a wavelength of 595 nm using a microtitre plate reader (BMG LABTECH GmbH, located in Allmendgrün, Germany).

### Assessment of antioxidant activity

The assessment of free radical scavenging activity (RSA) involved the measurement of decoloration in an ethanolic solution of DPPH radical, which was then examined using spectrophotometry at a wavelength of 517 nm [15]. The scavenging activity was calculated as follows:

$$\eqalign{&{\rm{Scavenging}}\,{\rm{ability }}\left( \%\right) \cr & = \left( {{{\rm{A}}_{{\rm{517}}\,{\rm{of}}\,{\rm{control}}}} - {{\rm{A}}_{{\rm{517}}\,{\rm{of}}\,{\rm{sample}}}}/{{\rm{A}}_{{\rm{517}}\,{\rm{of}}\,{\rm{control}}}}} \right) \times 100.}$$

### GC-MS analysis

According to the reported procedures [16], GC-MS analysis was conducted utilizing a Thermo Scientific Trace GC Ultra/ISQ Single Quadrupole MS instrument, equipped with a TG-5MS fused silica capillary column of 30 m in length, 0.251 mm in diameter, and featuring a 0.1 mm film thickness. The experimental setup involved the utilization of an electron ionization system with an ionization energy of 70 electron volts (eV). Helium gas was employed as the carrier gas, maintaining a consistent flow rate of 1 ml/min for gas chromatography-mass spectrometry (GC-MS) detection. The temperature of the MS transfer line was adjusted to 280 °C, while the injector was also set at the same temperature. The oven was initially set to a temperature of 50 °C and maintained at this level for a few minutes. Subsequently, the temperature was increased at a rate of 7 °C per minute until it reached 150 °C. Following this, the temperature was further increased at a rate of 5 °C per minute until it reached 270 °C. The oven was then held at this temperature for 2 min. Finally, the temperature was raised to a final value of 310 °C at a rate of 3.5 °C per minute and maintained at this level for a few minutes. The investigation involved quantifying all the discovered components through the use of a percent relative peak area. A preliminary determination of the compounds was conducted by comparing the relative retention time and mass spectra with the NIST and WILLY library data of the GC-MS instrument.

### *In silico* predictions of absorption, distribution, metabolism, and excretion (ADME)-related physicochemical properties and toxicity

The physicochemical features of the molecule were anticipated using the SwissADME web tools, which are specifically designed to assess ADME-related characteristics, Additionally, the toxicity of the selected compound has been predicted via ProTox II web server [17, 18].

### Statistical analysis

The data were presented as mean ± SE. Data obtained were analyzed by ANOVA one-way, t-test (*n* = 3 replicates) was used in comparisons.

## Results and discussion

### Microbial isolation

The fungus CO2 was isolated from the marine sponge *Corella cyathophora* (Fig. 1.a). The main morphological features were examined using a light microscope. The isolates exhibited notable differentiation when considering their morphological properties, including colony structure, texture, and medium pigmentation.

### Identification of the most potent fungal isolate

The morphological and microscopic inspection of the mycelium and spores cultivated on PDA plates provided confirmation that the isolate is classified as *Aspergillus* sp. based on the taxonomy established by [19] (Fig. 1.b and 1.c).

### Genetic identification of the associated fungus and bacteria

**Fig. 2:**
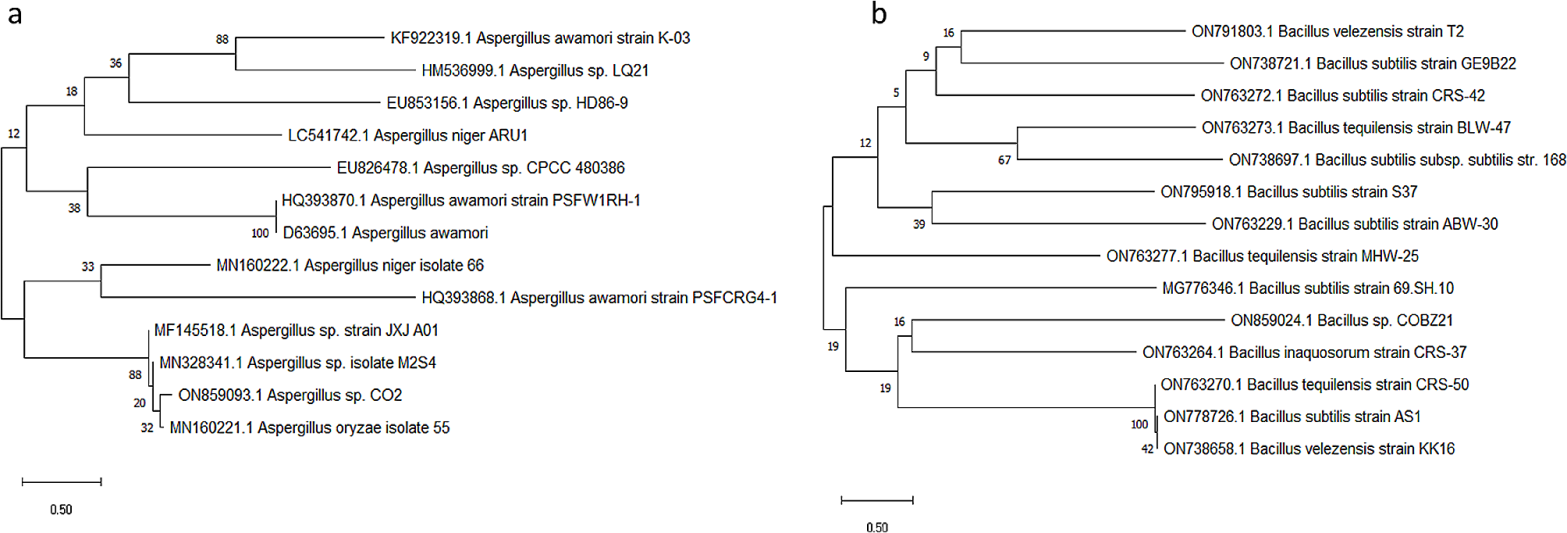
Phylogenetic trees of **(a)** *Aspergillus* sp. CO2 and **(b)** *Bacillus* sp. COBZ21

The genetic identification of the isolated fungus and bacteria was conducted by employing sequencing techniques targeting the 18 S rRNA and 16 S rRNA genes, respectively. The DNA was subjected to extraction, amplification, sequencing, and alignment with known sequences stored in the GeneBank database using the Basic Local Alignment Search Tool (BLAST). The results obtained demonstrated a high degree of similarity between the acquired sequence and the fungal isolate CO2, with a homology of 98.84% corresponding to *Aspergillus* sp. Similarly, the bacterial isolate COBZ21 exhibited a similarity of 99.68% with *Bacillus* sp. The identification of the CO2 isolate as *Aspergillus* sp. and the bacterial isolate as *Bacillus* sp. COBZ21 was determined through the examination of the DNA sequence and physical characteristics. The fungal and bacterial isolates have been archived in GenBank under the accession numbers ON859093.1 and ON859024.1, respectively. The evolutionary history was deduced by employing the neighbor-joining method, as proposed by Saitou and Nei [20]. The tree that demonstrates optimality is depicted. The bootstrap test was used to determine the percentage of duplicate trees in which the associated taxa grouped together [21]. These percentages are displayed adjacent to the branches. The illustrated tree has been accurately represented in terms of scale, where the lengths of the branches correlate to the evolutionary distances utilized in the inference of the phylogenetic tree (Fig. 2a and b). The Maximum Composite Likelihood technique was employed for determining the evolutionary distances and are in units of the number of base substitutions per site [10]. The current investigation included a total of 13 nucleotide sequences. This study included an analysis of the codon positions, which included the first, second, third, and noncoding locations. The paired deletion option was employed to remove all occurrences of ambiguous locations in each pair of sequences. The ultimate dataset comprised a total of 1755 sites. The software MEGA X was utilized to conduct evolutionary analysis [22].

### Metabolomic profiles of the fungal and bacterial axenic and co-culture extracts

Gas chromatography-mass spectrometry (GC-MS) analysis was employed to analyze the chemical profiles of the fungi *Aspergillus* sp. CO2 and *Bacillus* sp. COBZ21 (Figs. 1 and 2S) following their fermentations, both under axenic and co-fermentation conditions. The metabolomic profile of the co-culture extract exhibited the stimulation of various metabolites from distinct chemical classes in contrast to those observed in the two separate cultures. The total peak areas of the discovered constituents in the axenic culture of *Bacillus* sp. amount to 40.08%. The chemical structures of these identified compounds are documented in Table 1. The main detected compounds are (+-) cis-Deethylburnamine (2.66%) and 2,7,12,17-Tetraethyl-3,5:8,10:13,15:18,20-tetrakis (2,2-di methylpropano) porphyrin (2.60%). Moreover, the analysis of *Aspergillus* sp. CO2 extract using gas chromatography-mass spectrometry (GC-MS) resulted in the detection and identification of a total of 28 components (Table 2). The total peak areas of the identified ingredients constitute 49.56%, the prospects of the chemical structures of the identified compounds are recorded in Table 2. The main detected compounds are Bis(3,6,9,12-tetraoxapentaethylene)crowno-N,N,N’,N’-tetra methylpphanediamine (2.48%), Lipo-3-episapelin A (2.52%), 2,3-Diacetoxy-6,7,10,11-tetrapentyloxytriphenylene (2.27%), and 3-(2-Dimethylaminoethyl)-5-hydroxy-4-(3-hydroxypropyl)indole (2.23%). The study of *Aspergillus* sp. CO2 and *Bacillus* sp. COBZ21 coculturing extracts by GC-MS resulted in the discovery of 28 compounds, as shown in Table 3. The cumulative peak areas of the detected components account for 55.98% of the total. The chemical structures of these identified compounds are documented in Table 3. The main detected compounds are 11-phenyl-2,4,6,8-tetra(2-thienyl)-11-aza-5,13-dithiaeteracyclo[7.3.0.1(2,8)0.0(3,7)]trideca-3,6-diene-10,12,13-trione (3.13%), 2-[3’,5’-di(t-Butyl)-4’-hydroxyphenyl)-3-methyl-1,4-naphthoquinone (3.04%), 21-(1-methoxycarbonylethyl)-à,á,.delta.,ç-tetramethyl porphyrine (2.68%). The mixed fermentation of *Aspergillus* sp. CO2 and *Bacillus* sp. COBZ21 resulted in the formation of various chemicals, as indicated by the chemical structure composition analysis (refer to Table 3). The observed induction may arise from either environmental competition or chemical defense systems. Following that, the samples obtained from two pure cultures and a mixed culture were analyzed for their antibacterial, antibiofilm, and antioxidant properties.

**Fig. 3:**
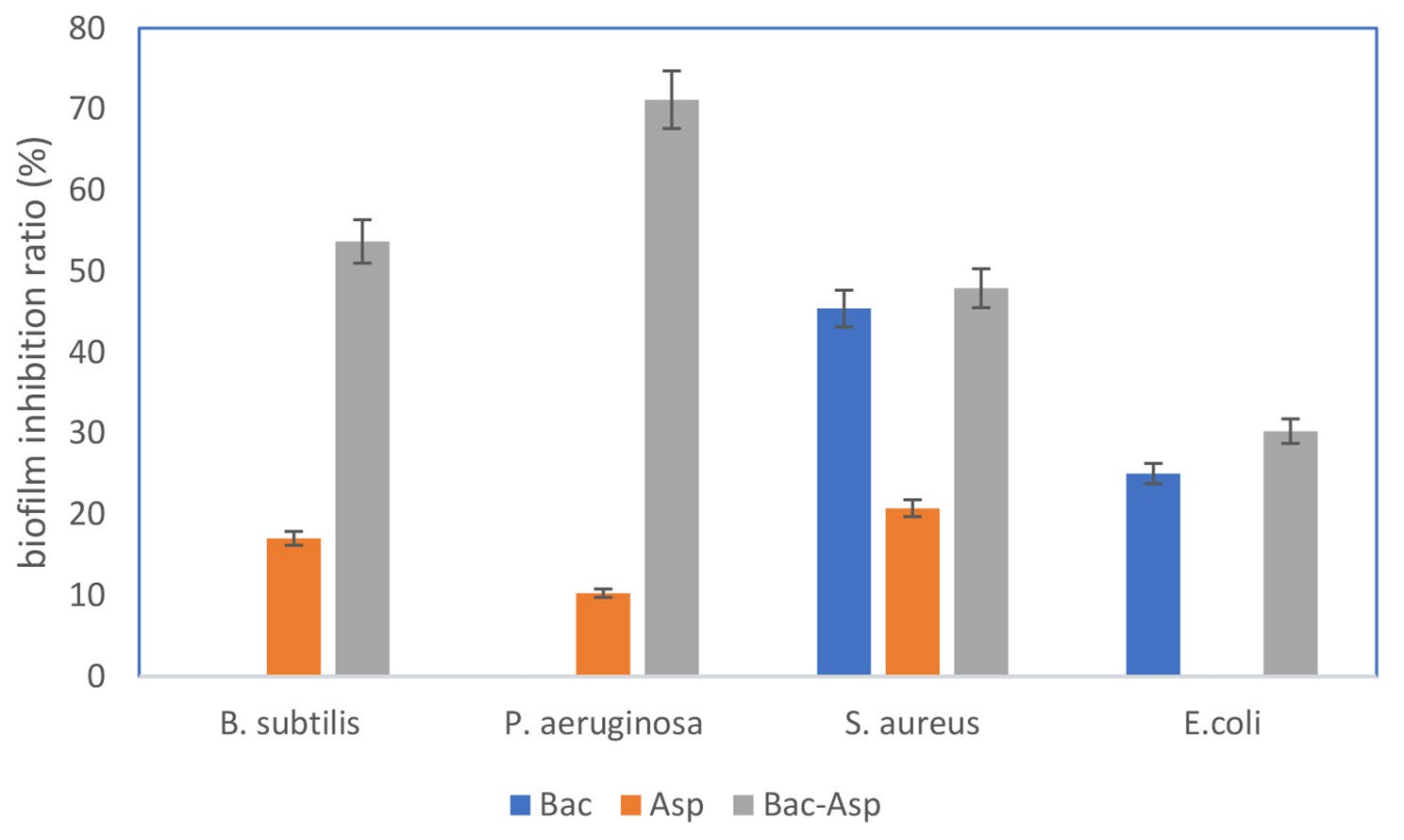
Biofilm inhibitory activity of bacterial, fungal and mixing culture **Bac**: *extract of Bacillus* sp. COBZ21, **Asp**: *extract of Aspergillus sp.* CO2 & **Ba-Asp**: *extract of coculture between Bacillus* sp. COBZ21 *and Aspergillus sp.* CO2 ***B. subtilis***: *Bacillus****subtilis*ATCC 6633**, ***P. aeruginosa***: *Pseudomonas aeruginosa* ATCC 10,145, ***S. aureus***: *Staphylococcus aureus* NRRLB-767, ***E. coli***: *Escherichia coli* ATCC 25,922

**Table 1:** Chemical compositions of *Bacillus* sp. COBZ21

No.	R_t_	Area%^a^	M.W.	M.F.	Identified compounds
1	7.40	1.94	538	C_34_H_50_O_5_	1’,1’-Dicarboethoxy-1á, 2á-dihydro-3’H-cycloprop [1, 2]cholesta-1,4,6-trien-3-one
2	7.79	1.60	641	C_32_H_19_NO_2_S_6_	6(N)-phenyl-1,3,4,8-tetra(2’-thienyl)5,7-dioxo-4,8-epithio-thieno[3,4-f]isoindole
3	8.42	1.66	616	C_40_H_44_N_2_O_4_	6,15,33,42-Tetraoxa-43,46-diazaheptacyclo[18.12.10.4 (22,31)0.0(5,32)0.0(16,21)0.0 (25,45)0.0 (28,44)] hexatetraconta-1,3,5(32),16,18,20,22,24,26,28,30,43,45-tridecaene
4	9.66	1.77	679	C_42_H_37_N_3_O_6_	4,6-Dimethoxy-7-(4’,6’-dimethoxyI-7’-(4”,6”-dimethoxy indol-2”-yl)indol-2’-yl)-2,3-diphenylindole
5	10.64	2.03	656	C_42_H_72_O_5_	Lipo-3-episapelin A
6	12.22	2.03	618	C_39_H_54_O_6_	2-Hydroxy-3-methoxy-6,7,10,11-tetrapentyloxytriphenylene
7	12.48	1.68	214	C_7_H_10_N_4_O_2_S	6-Hydroxymoethyl-5,6,7,8-tetrahydro-5-deaza-5-thiapterin
8	13.59	2.60	694	C_48_H_62_N_4_	2,7,12,17-Tetraethyl-3,5:8,10:13,15:18,20-tetrakis(2,2-di methylpropano)porphyrin
9	19.08	1.90	612	C_48_H_36_	5,6,8,9,21,22,24,25-Octahydro[2.2](3,11)dibenzo[a,j]anthraxcenophane-1,17-diene
10	31.26	1.97	509	C_30_H_23_NO_7_	2-Hydroxy-2-[1’-(2”,3”-dihydro-2”-hydroxy-1”,3”-dioxo-1H-inden-2”-yl)-2’-morpholino-2’-phenyethenyl]-1 H-inden e-1,3(2 H)-dione
11	31.41	1.80	379	C_22_H_41_N_3_O_2_	(3R,4R)-3,4-Bis(3,3-dimethylbutylamido)-1-cyclohexyl pyrrolidine
12	31.75	2.66	250	C_17_H_18_N_2_	(+-)cis-Deethylburnamine
13	33.06	1.59	652	C_37_H_40_N_4_O_7_	2-Formyl-4,6,7-tris(2-methoxycarbonylethyl)-1,3,5,8-tetra methylporphin
14	34.72	1.83	256	C_17_H_36_O	1-Heptadecanol
15	37.45	1.89	562	C_35_H_46_O_6_	2-Hydroxy-3-methoxy-6,7,10,11-tetrabutyloxytriphenylene
16	39.95	1.75	398	C_29_H_50_	24-ethyl-cholest-ene
17	40.48	1.68	277	C_17_H_11_NO_3_	7-Methoxy1azatetracyclo[8.7.0.0(4,9)0.0(12,17)]heptadeca-2,4,7,9,12(17),13,15-octaen-6,11-dione
18	40.77	2.17	689	C_45_H_31_N_5_O_3_	2-Methoxy-3-nitro-5,10,15,20-tetraphenyl-2,3-dihydroporphyrin
19	42.35	2.03	692	C_44_H_44_N_4_O_4_	N,N’-Dicyclohexyl-1,7-dipyrrolidinylperylene-3,4:9,10-tetra carboxylic acid bisimide
20	43.55	1.84	528	C_33_H_54_NO_4_	3’-(Cholan-3-one-24-oate)-2’,2’,5’,5’-tetramethylpyrrolidine-1’-oxyl
21	47.48	1.66	696	C_40_H_56_O_10_	Nephthoside-1,2’,3’,4’-Tetraacetate
		**T% 40.08**			

**Rt**: Retention time; **M.W.**: Molecular weight; **M.F.**: Molecular formula

**Table 2:** Chemical compositions of *Aspergillus* sp. CO2

No.	R_t_	Area %^a^	M.W.	M.F.	Identified compounds
1	8.28	1.73	692	C_44_H_44_N_4_O_4_	N,N’-Dicyclohexyl-1,7-dipyrrolidinylperylene-3,4:9,10-tetracarboxylic acid bisimide
2	9.08	1.85	694	C_48_H_62_N_4_	2,7,12,17-Tetraethyl-3,5:8,10:13,15:18,20-tetrakis(2,2-dimethylpropano)porphyrin
3	9.32	1.73	658	C_45_H_42_N_2_O_3_	3,5-DitButyl-4-hydroxyphenylbis(1,2-dihydro-2-oxo-N-phenylcyclohepta[b]pyrrol-3 yl)methane
4	9.52	2.27	618	C_37_H_38_N_4_O_5_	34,38-Dioxo-35,36,37-trimethoxy-3,7,23,27-tetraazahepta cyclo-(27.3.0.1.1.1.1.1.1) ocataconta1(33),8(35),9,11,13-(36),14,16,18(37),19,21,29,31-dodecaene
5	12.58	1.77	634	C_38_H_42_N_4_O_5_	2,2’’-Dimethoxy-2’-(phenylmethoxy)-3,3’’-bis(hexahydro-2-oxopyrimidinyl)-1,1’:3’,1’’terphenyl
6	13.02	1.87	217	C_13_H_15_NO_2_	3a,4-cis-3a,6a-cis-3-(4-methylphenyl)-3a,5,6,6a-tetrahydro -4 H-cyclopenta[d]isoxazole-4-ol
7	15.51	2.03	315	C_17_H_21_N_3_OS	2-Phenyl-3-amethyl-3-thioxoimidazolidin[1,5a]transperhydroquinoxalin-4-one
8	19.65	1.96	696	C_46_H_52_N_2_O_4_	2-[2,6-Bis(hex-5-enyloxy)phenyl]9[2-(but-3-enyloxy)-6-(hex-5-enyloxy)phenyl]-1,10-phenanthroline
9	22.12	1.72	610	C_36_H_42_N_4_O_5_	2(4)-ethyl-4(2)-(1-hydroxyethyl)deuteroporphyrin dimethyl ester
10	26.44	2.18	662	C_39_H_42_N_4_O_6_	34,38-Dioxo-33,35,36,37-tetramethoxy-31-methyl-3,7,23,27-tetraazaheptacyclo (27.3.1.1.1.1.1.1) octaconta-1-(33),8-(35),9,11,13(36),14,16,18(37), 19,21,29,31-dodecaene
11	27.15	1.76	369	C_21_H_23_NO_5_	(R)-O-[2-(4-Methoxyphenylamino)phenyl]acetylpantolactone
12	28.73	2.52	656	C_42_H_72_O_5_	Lipo-3-episapelin A
13	32.22	1.84	176	C_8_H_16_O_2_S	trans-1-Methoxy-2-methylsulfinylcyclohexane
14	32.67	2.11	207	C_7_H_13_NO_2_S_2_	N-[Bis(methylthio)methylene]-L-alaninemethyl ester
15	34.92	1.88	691	C_51_H_33_NO_2_	2,6-Bis(2,3,5-triphenyl-4-oxocyclopentadienyl)pyridine
16	39.60	1.98	708	C_44_H_36_O_9_	3,5-Diphenyl-3,5-(9,10-phenanthylene)tricyclo[5.2.1.0]decane-4-one-8-exo-9-endodicarboxylic acid diacetoxy methylester
17	41.67	1.88	562	C_40_H_50_O_2_	Rhodoxanthin
18	43.86	1.78	328	C_20_H_24_O_4_	Dimethyl 3,4,8-trimethyl-6-propylazulene-1,2-dicarboxylate
19	46.63	1.90	412	C_24_H_36_N_4_S	2-Pentadecyl-1,2,4-triazolo[1,5c]quinazoline-5-(6 H)-thione
20	47.34	1.96	110	C_5_H_6_N_2_O	Pyrazine, methyl-, 1-oxide
21	48.02	1.89	696	C_40_H_56_O_10_	Nephthoside 1,2’,3’,4’-Tetraacetate
22	48.55	2.48	676	C_36_H_60_N_4_O_8_	Bis(3,6,9,12-tetraoxapentaethylene)crowno-N,N,N’,N’-tetra methylpphanediamine
23	49.80	1.97	732	C_42_H_44_N_4_O_8_	13-(Ethylidenedioxy)cyclohexadieno[1,6-b]phylloerythrin trimethyl ester
24	50.09	2.27	688	C_42_H_56_O_8_	2,3-Diacetoxy-6,7,10,11-tetrapentyloxytriphenylene
25	52.57	2.23	262	C_15_H_22_N_2_O_2_	3-(2-Dimethylaminoethyl)-5-hydroxy-4-(3-hydroxypropyl)indole
		**T% 49.56**			

**Rt**: Retention time; **M.W.**: Molecular weight; **M.F.**: Molecular formula

**Table 3:** Chemical compositions of *Aspergillus* sp. CO2 and *Bacillus* sp. COBZ21 coculturing

No.	R_t_	Area%^a^	M.W.	M.F.	Identified compounds
1	12.30	2.68	700	C_48_H_36_N_4_O_2_	21-(1-methoxycarbonylethyl)-à,á,.delta.,ç-tetramethyl porphyrine
2	13.58	1.99	335	C_20_H_21_N_3_O_2_	N(2)-BenzylN(2)-methyl-3-(2-hydroxypropyl)-2-quinoxalinecarboxamide
3	14.34	2.25	356	C_18_H_16_N_2_O_6_	1-carbethoxy-3,4-dicarbomethoxy-ç-carboline
4	15.09	1.68	621	C_27_H_43_NO_11_S_2_	13,14-Bis(methylsulfonyl)-2-dehydro-3-dehydroxy pseudaconine
5	15.29	3.04	376	C_25_H_28_O_3_	2-[3’,5’-di(t-Butyl)-4’-hydroxyphenyl)-3-methyl-1,4-naphthoquinone
6	15.53	1.86	730	C_55_H_70_	Tris(3,6-di-t-butyl-1-azulenyl)methane
7	16.14	1.67	612	C_40_H_40_N_2_O_4_	2-(2’,6’-Dimethoxyphenyl)-4,7-bis[4’-(1”,1”-dimethyl)phenoxy]1,10-phenanthroline
8	16.73	2.03	688	C_42_H_56_O_8_	2,3-Diacetoxy-6,7,10,11-tetrapentyloxytriphenylene
9	17.48	1.65	704	C_33_H_36_O_17_	6-C-Xylosyl-8-C-glucosylapigenin permethylated derivative
10	17.58	1.65	692	C_44_H_44_N_4_O_4_	N,N’-Dicyclohexyl-1,7-dipyrrolidinylperylene-3,4:9,10-tetracarboxylic acid bisimide
11	17.83	2.48	696	C_38_H_48_S_6_	2,3,6,7,10,11-Hexakis(ethylsulfanyl)-4b,8b,12b,12d-tetra methyl-4b,8b,12b,12d tetrahydrodibenzo [2,3:4,5]pentaleno[1,6ab]indene
12	18.64	1.87	691	C_51_H_33_NO_2_	2,6-Bis(2,3,5-triphenyl-4-oxocyclopentadienyl)pyridine
13	25.32	1.62	689	C_45_H_31_N_5_O_3_	2-Methoxy-3-nitro-5,10,15,20-tetraphenyl-2,3-dihydroporphyrin
14	32.40	1.62	686	C41H66O8	(2R)-8,13-epoxy-2,2-(8’,13’-epoxy-2’b-methoxy-3’-oxolabdane-1’a,2’a-diyldioxy)-1a-hydroxylabdan-3-one
15	32.62	2.23	696	C_40_H_56_O_10_	Nephthoside-1,2’,3’,4’-Tetraacetate
16	32.79	1.53	605	C_38_H_47_N_5_O_2_	meso-1-(2’-ethoxycarbonyl-2’-cyanoethyl)-3,6,9,12-tetramethylporphyrin
17	35.25	1.77	696	C_44_H_28_N_10_	2,3,5,6-tetrakis[6’-(2’’,2’’’-Bipyridyl)]pyrazine
18	37.28	1.97	221	C_10_H_11_N_3_O_3_	2-Isopropoxy-3-nitroimidazo[1,2a]pyridine
19	40.40	1.98	485	C_34_H_47_NO	(R)(+)4-Benzyl-1-(4-methoxyphenyl)-4-phenylaminotetradecane
20	43.73	2.04	584	C_40_H_56_O_3_	Antheraxanthin
21	44.02	1.83	658	C_45_H_42_N_2_O_3_	3,5-Di-t-Butyl-4-hydroxyphenylbis(1,2-dihydro-2-oxo-N-phenylcyclohepta[b]pyrrol-3-yl)methane
22	44.84	1.82	681	C_42_H_39_N_3_O_6_	Triethyl 3,7,11triphenylcyclonona[1,2b:4,5b’:7,8b”]tripyrrole-2,6,10-tricarboxylate
23	46.61	2.0	572	C_30_H_36_O_11_	Carda-16,20(22)-dienolide,3-[(6-deoxy-3,4-O-methylene hexopyranos-2-ulos-1-yl)oxy]-7,8-epoxy-11,14-dihydroxy-12-oxo,(3á,5á,7á,11à)
24	47.24	1.53	644	C_44_H_32_N_6_	5,10-bis(3-aminophenyl)-15,20-diphenylporphyrin
25	47.61	1.53	733	C_37_H_67_NO_13_	Erythromycine
26	47.95	3.13	657	C_32_H_19_NO_3_S_6_	11-phenyl-2,4,6,8-tetra(2-thienyl)-11-aza-5,13-dithiaeteracyclo[7.3.0.1(2,8)0.0(3,7)]trideca-3,6-diene-10,12,13-trione
27	49.40	2.32	694	C_48_H_62_N_4_	2,7,12,17-Tetraethyl-3,5:8,10:13,15:18,20-tetrakis(2,2-dimethylpropano)porphyrin
28	53.0	2.21	708	C_44_H_36_O_9_	3,5-Diphenyl-3,5(9,10- phenanthylene)tricyclo[5.2.1.0] decane-4-one-8-exo-9-endodicarboxylic acid
		**T% 55.98**			

**Rt**: Retention time; **M.W.**: Molecular weight; **M.F.**: Molecular formula

### Biological evaluation

#### Antimicrobial activity

Antimicrobial resistance (AMR) arises as a consequence of the evolutionary adaptation of bacteria, viruses, fungi, and parasites, resulting in the loss of their capacity to effectively react to antibiotics. This phenomenon renders infections more challenging to manage, amplifying the likelihood of disease transmission, severe morbidity, and mortality. The efficacy of antibiotics and other antimicrobial treatments is compromised due to the emergence of drug resistance, rendering the treatment of illnesses increasingly challenging or even unattainable [12]. The antimicrobial activity of the three extracts was evaluated against pathogenic bacteria and fungi using 96-well flat polystyrene plates. The obtained results revealed that the bacterial extract displayed moderate antibacterial activity against *S. aureus* NRRLB-767 and *E. coli* ATCC 25,922. Furthermore, the fungal extract (ASP) showed low antibacterial activity against *Klebsiella, Salmonella*, and *S. aureus* NRRLB-767, while it displayed pronounced activity against *E. coli* ATCC 25,922. Additionally, the co-culturing extract (Ba-Asp) exhibited potent antibacterial activity against *E. coli* ATCC 25,922 and *S. aureus* NRRLB-767, while it showed low antibacterial activity against *Salmonella and Klebsiella* and antifungal activity against *C. albicans* ATCC 10,231 (Table 4). The MIC of three extracts was measured toward positive strains and presented in Table 5. The study conducted by Moubasher et al., [23] showed that the co-culturing of fungi and bacteria holds promise as a viable approach for generating secondary metabolites that possess antibacterial properties. The findings from the co-culture experiment revealed that bacteria subjected to chemical stress exhibited diverse reactions, leading to the production of fungal secondary metabolites that possess antibacterial properties. During the co-culture of eight Streptomyces strains and nine fungi, Nicault et al., [24] examined a total of 72 distinct interaction zones involving Streptomyces-fungus interactions (SFIZs). Two of the samples were chosen due to their demonstration of an increase in antibacterial activity when compared to the individual cultures. The examination of these SFIZs revealed that co-cultivation exerted a significant influence on the metabolic expression of each participant, hence facilitating the production of distinct chemicals. The findings indicated that investigating the metabolic capabilities of Streptomyces and fungi through the replication of biotic interactions observed in this particular ecological niche holds considerable potential as a research direction.

**Table 4:** Antimicrobial activity of different extracts

Sample	Inhibition ratio (%)						
	Kle	Sal	Sta	MRSA	Ech	Can	Asper
**Bac**	NA	NA	50.50	NA	20.25	NA	NA
**ASP**	12.25	11.02	22.20	NA	44.011	NA	NA
**Ba-Asp**	23.00	15.00	65.00	NA	75.00	50.00	NA
**Nys**	-	-	-	-	-	97	98
**Cip**	98	-	96	-	98	-	-

**Bac**: *extract of Bacillus* sp. COBZ21, **Asp**: *extract of Aspergillus sp.* CO2 & **Ba-Asp**: *extract of coculture between Bacillus* sp. COBZ21 *and Aspergillus sp.* CO2. *&***Nys**: Nystatin & **Cip**: Ciprofluxacin

**Kle**: *Klebsiella pneumoniae*, **Sal**: *Salmonella typhi*, **Sta**: *Staphylococcus aureus* NRRLB-767, **Ech**: *Escherichia coli* ATCC 25,922, **Can**: *Candida albicans* ATCC 10,231, **Asper**: *Aspergillus niger* ATCC 10,231

**Table 5:** Minimum inhibitory concentration different extracts

Sample	MIC (µg/mL)			
	Kle	Sta	Ech	Can
**Bac**	-	31.50	125	-
**ASP**	250	125.00	31.25	-
**Ba-Asp**	125	15.25	7.25	50.00
**Nys**		-	-	0.390
**Cip**	-	3.50	0.390	-

**Bac**: *extract of Bacillus* sp. COBZ21, **Asp**: *extract of Aspergillus sp.* CO2 & **Ba-Asp**: *extract of coculture between Bacillus* sp. COBZ21 *and Aspergillus sp.* CO2. *&***Nys**: Nystatin & **Cip**: Ciprofluxacin

**Kle**: *Klebsiella pneumoniae*, **Sta**: *Staphylococcus aureus* NRRLB-767, **Ech**: *Escherichia coli* ATCC 25,922, **Can**: *Candida albicans* ATCC 10,231

### Antibiofilm assay

Bacterial biofilms are intricate assemblages of bacteria that adhere to surfaces and are held together by matrices composed primarily of polysaccharides, proteins, and extracellular DNA [25]. Bacterial biofilms have been identified as significant contributors to bacterial persistence and are a source of nosocomial infection that is engaged in a variety of infectious illnesses [26]. The antibiofilm efficacy of the extracts obtained from both axenic and coculture conditions was evaluated through the utilization of the MTT test against four distinct pathogenic bacterial strains, including *P. aeruginosa* ATCC 10,145, *S. aureus* NRRLB-767, *E. coli* ATCC 25,922, *and B. subtilis* ATCC 6633. The obtained results showed that the bacterial extract (BAC) has low to moderate antibiofilm activity toward *S. aureus* NRRLB-767 and *E. coli* ATCC 25,922 *only*, with biofilm inhibition ratios of up to 45.38 and 25.02%, respectively, while the fungal extract (Asp) displayed very low biofilm inhibition activity against *B. subtilis* ATCC 6633, *P. aeruginosa* ATCC 10,145, and *S. aureus* NRRLB-767, with biofilm inhibitory activity reaching 17.02, 10.25, and 20.73%, while no activity was measured *against E. coli* ATCC 25,922. On the other hand, mixed culture (Bac-Asp) showed significant biofilm inhibitory activity toward *B. subtilis* ATCC 6633, *P. aeruginosa* ATCC 10,145, and *S. aureus* NRRLB-767, *with activity up to 53.66*, 71.17, *and* 47.89*%*, while it showed low activity against *E. coli* ATCC 25,922 (Fig. 3).

### Antioxidant activity

The disparity between the protective capacity of antioxidants and the generation of reactive species is a significant concern, as it contributes to the development of various diseases by inducing harm to the genome and other macromolecules. There is a significant amount of literature indicating that increased intake of antioxidants through dietary supplementation is associated with a reduced likelihood of developing a range of diseases. Furthermore, the inclusion of nutraceuticals containing natural chemicals, particularly antioxidants, is vital within the realm of food products [27]. So, in this study, the antioxidant activity of bacterial, fungal, and mixed extracts based on the DPPH assay (200 µg/ml) showed the most antioxidant agent, showing maximum DPPH scavenging activity (75.25%), followed by fungal extract alone (60.26%), then bacterial extract (41.22%) (Table 6). Study on the antioxidant activity of a coculture extract derived from the mycelia of *Ganoderma lucidum* and *Flammulina velutipes* in a submerged fermentation process. The assessment of antioxidant activity was conducted using the quantification of ABTS radical scavenging activity. The IC_50_ values for the extracts of *G. lucidum* and *F. velutipes*, as well as their monocultures and cocultures, were determined to be 1.54, 1.99, and 1.64 mg/mL, respectively. The aforementioned findings substantiated that the monoculture of *G. lucidum* exhibited superior antioxidant activity in comparison to the monoculture of *F. velutipes*. Furthermore, the coculture showed antioxidant activity that was equal to that of *G. lucidum* monoculture [28]. Sutthiphatkul, [29] attempts to determine the most favorable co-culture ratio of *Acetobacter pasteurianus* AJ605 and *Zygosaccharomyces bailii* YN403 for the fermentation of kombucha. The findings of the study indicate that the maximum antioxidant activity was observed when a co-culture ratio of 8:2 (v/v) of *A. pasteurianus* AJ605 and *Z. bailii* YN403 was used over a 10-day fermentation period. The resulting DPPH IC_50_ value was measured at 25.76 µl/mL, while the ABTS IC_50_ value was determined to be 8.84 µl/mL. The antioxidant activity of the co-culture ratio of 8:2 was found to be statistically indistinguishable (*p* > 0.05) from that of the symbiotic culture of bacteria and yeasts (SCOBY) alone.

**Table 6:** DPPH scavenging activity (%) of different extracts

Sample	Time (min.)			
	15	30	45	60
Bac	10.05	19.26	25.26	41.22
Asp	15.58	29.87	42.96	60.26
Bac-Asp	22.02	45.25	59.55	75.25

**Bac**: *extract of Bacillus* sp. COBZ21, **Asp**: *extract of Aspergillus sp.* CO2 & **Ba-Asp**: *extract of coculture between Bacillus* sp. COBZ21 *and Aspergillus sp.* CO2

#### The ADME-related physicochemical properties

**Fig. 4:**
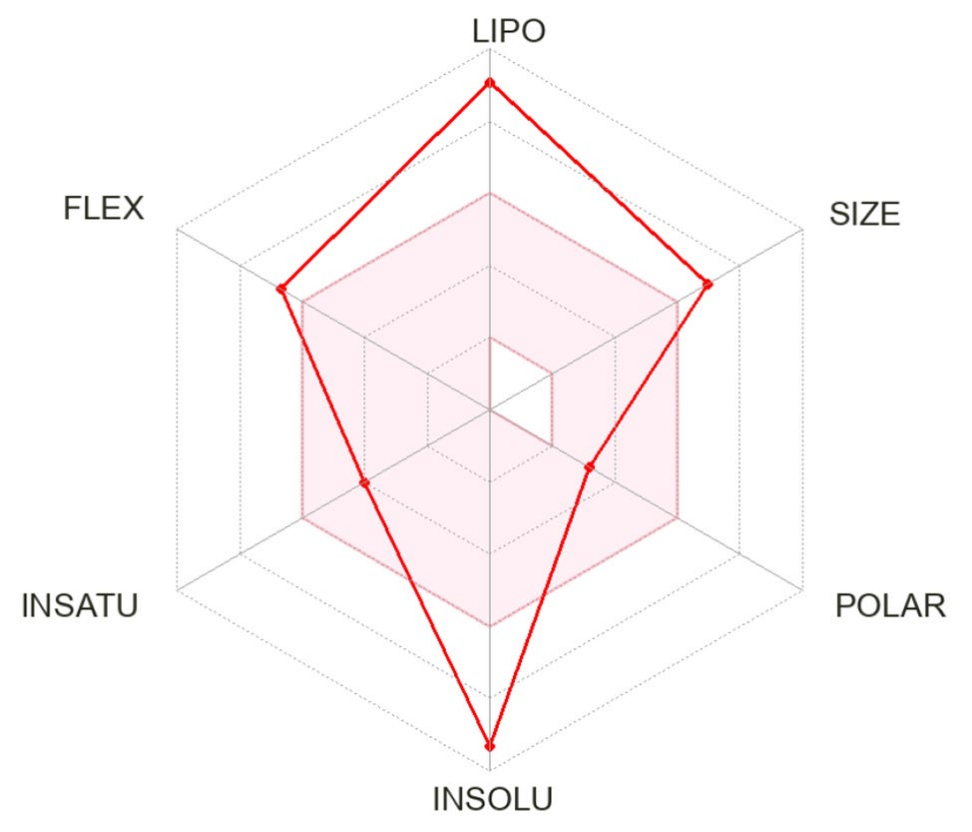
Bioavailability Radar plot of Ba-Asp The pink area shows the optimal range for each property (Lipophilicity: XLOGP3 between − 0.7 and + 5.0, size: MW between 150 and 500 g/mol, polarity: TPSA between 20 and 130 Å^2^, solubility: log S not higher than 6, saturation: fraction of carbons in the sp^3^ hybridization not less than 0.25, and flexibility: no more than 9 rotatable bonds)

Based on the GC-mass analysis of the mixed culture of *Aspergillus* sp. CO2 and *Bacillus* sp. COBZ21, Physicochemical properties of the first compound 11-phenyl-2,4,6,8-tetra(2-thienyl)-11-aza-5,13-dithiaeteracyclo[7.3.0.1(2,8)0.0(3,7)]trideca-3,6-diene- 0,12,13-trione was detected using the SwissADME online server [18]. The estimations are based on drug-likeness guidelines. Accordingly, the compound did not pass the Lipinski du due to two violations (MW > 500, MLOGP > 4.15), Ghose rules, due to four violations (MW > 480, WLOGP > 5.6, MR > 130, #atoms > 70). The compound exhibited 0.17% oral bioavailability, which reflects its poor properties as an oral medication (Table 7). Furthermore, the expeditious assessment of drug-likeness was conducted by the graphical representation of bioavailability. A radar map was generated in order to assess and compare the two compounds using six physicochemical properties, namely size, polarity, lipophilicity, solubility, flexibility, and saturation. The depicted region in the diagram corresponds to the optimal range of values for each parameter. Based on the diagram provided, the compound exhibited an optimal range (shown by the pink area) for all criteria, with the exception of size, flexibility, insolubility, and lipophilicity (Fig. 4). Lipophilicity, an additional physicochemical property of significance, serves as an indicator of the compound’s capacity to permeate the cell membrane [30, 31]. The compound showed Log *P*_o/w_ values above 5 (8.82), suggesting bad permeability and absorption across the cell membrane. Moreover, the solubility of a molecule is a critical factor that significantly impacts the absorption of the compound throughout the formulation process [18]. According to the ESOL topological model, it may be inferred that the chemical exhibits a modest level of solubility. In order to provide a comprehensive definition of medicinal chemistry and leadlikness, it is worth noting that the molecule in question did not meet the criteria outlined by the rule of three (RO3). Table 8 shows the compound pharmacokinetic parameters measured using the vector machine algorithm (SVM) model [18].

**Table 7:** ADME-related physicochemical parameters of Ba-Asp

Predictive models parameters		Compound 1
Physicochemical Properties	Molecular Weight	584.87 g/mol
	Fraction Csp3	0.50
	Rotatable bonds	10
	H-bond acceptors	3
	H-bond donors	2
	Molar Refractivity	186.28
	Topological polar surface area (TPSA)	52.99 Å²
Lipophilicity	Log Po/w (XLOGP3)	10.34
	Log *P*_o/w_ (WLOGP)	9.76
	Log *P*_o/w_ (MLOGP)	6.15
Solubility	Log *S* (ESOL)	-5.66
	Solubility	2 1.28e-03 mg/ml; 2.18e-06 mol/l
	Class	Moderately soluble
Druglikeness	Lipinski (RO5)	No; 2 violations: MW > 500, MLOGP > 4.15
	Ghose	No; 4 violations: MW > 480, WLOGP > 5.6, MR > 130, #atoms > 70
	Veber	Yes
	Bioavailability Score	0.17
Leadlikness	Rule of three (RO3)	No; 3 violations: MW > 350, Rotors > 7, XLOGP3 > 3.5
	Synthetic accessibility	7.37

Log P_o/w_ = The partition coefficient between n-octanol and water, Log S = The decimal logarithm of the molar solubility in water. ^f^ Lipinski (RO5) criteria range are lipophilicity (Log P_o/w_) ≤ 5, MW ≤ 500, H-bond donors ≤ 5, and H-bond acceptors ≤ 10. Ghose filter criteria range is Log P_o/w_ in -0.4 to + 5.6 range, MR from 40 to 130, MW from 180 to 480, No. of atoms from 20 to 70. Veber rule criteria range are: RB ≤ 10 and TPSA ≤ 140 Å^2^. ^i^RO3 criteria range is XLOGP3 ≤ 3.5, MW ≤ 350, H-bond donors ≤ 3, H-bond acceptors ≤ 3, and RB ≤ 3. Synthetic accessibility (SA) score ranges from 1 (very easy) to 10 (very difficult)

**Table 8:** Pharmacokinetics parameters of Ba-Asp

Pharmacokinetics Parameters	C1
GI (HIA) absorption	Low
BBB permeant	No
P-gp substrate	Yes
CYP1A2 inhibitor CYP2C19 inhibitor CYP2C9 inhibitor	No
	No
	No
CYP2D6 inhibitor	No
CYP3A4 inhibitor	No
Log *K*_*p*_ (skin permeation: cm/s)	-2.53 cm/s

GI (HIA) = Human gastrointestinal absorption, BBB = Blood-brain barrier permeation P-gp = Permeability glycoprotein, Log K_p_ = The skin permeability coefficient

### Toxicity prediction via ProTox II

**Fig. 5:**
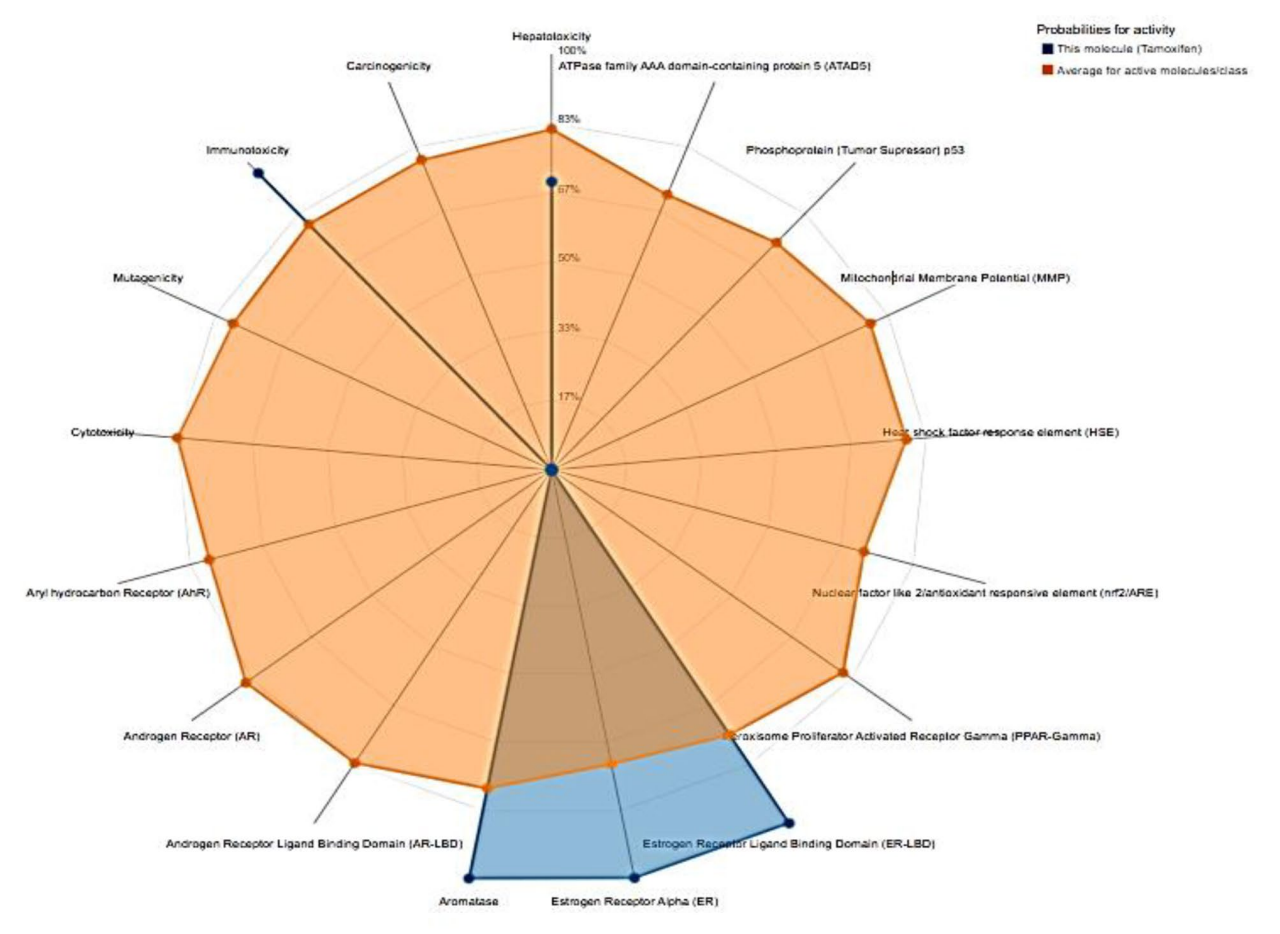
Toxicity radar chart

Using the ProTox II website, the compound’s toxicity prediction was carried out [32]. Results in Table 9 showed that, the selected compound has no pronounced toxicity, as predicted by ProTox II. The primary purpose of the toxicity radar chart is to provide a concise visual representation of the level of certainty associated with positive toxicity outcomes in relation to the average value within each respective category (Fig. 5).

**Table 9:** *In silico* toxicity prediction of Ba-Asp compound

Classification	Target	Ba-Asp
Organ toxicity	Hepatotoxicity	Active
Toxicity end points	Carcinogenicity	Inactive
	Immunotoxicity	Active
	Mutagenicity	Inactive
	Cytotoxicity	Inactive
Tox21-Nuclear receptor signaling pathways	Aryl hydrocarbon Receptor (AhR)	Inactive
	Androgen Receptor (AR)	Inactive
	Androgen Receptor Ligand Binding Domain (AR-LBD)	Inactive
	Aromatase	Active
	Estrogen Receptor Alpha (ER)	Active
	Estrogen Receptor Ligand Binding Domain (ER-LBD)	Active
	Peroxisome Proliferator Activated Receptor Gamma (PPAR-Gamma)	Inactive
Tox21-Stress response pathways	Nuclear factor (erythroid-derived 2)-like 2/antioxidant responsive element (nrf2/ARE)	Inactive
	Heat shock factor response element (HSE)	Inactive
	Mitochondrial Membrane Potential (MMP)	Inactive
	Phosphoprotein (Tumor Supressor) p53	Inactive
	ATPase family AAA domain-containing protein 5 (ATAD5)	Inactive

## Conclusion

The fungal species *Aspergillus* sp. CO2 and the bacterial species *Bacillus* sp. COBZ21 have been obtained by isolation procedures from the sea sponge *Corella cyathophora*. Gas chromatography-mass spectrometry (GC-MS) was employed to analyze the chemical profiles of the crude extracts derived from *Aspergillus* sp. CO2, *Bacillus* sp. COBZ21, and their coculture. The employment of a coculture approach including *Bacillus* sp. COBZ21 and *Aspergillus* sp. CO2 resulted in a notable augmentation of many activities, including antibacterial, antibiofilm, and antioxidant properties. Subsequently, utilizing gas chromatography-mass spectrometry examination of the heterogeneous culture the ADME-related physicochemical parameters of the first drug were evaluated utilizing the SwissADME web server. The toxicity assessment of the coculture extract was conducted using the ProTox II web site, which indicated that the chosen chemical does not exhibit significant toxicity.

### Electronic supplementary material

Below is the link to the electronic supplementary material.

Supplementary Material 1

## Data Availability

The datasets generated and/or analyzed during the current study are available in the GenBank repository https://www.ncbi.nlm.nih.gov, [Accession numbers ON859093.1 and ON859024.1].

## References

[1] Patil RH, Patil MP, Maheshwari VL. Chapter 5 - bioactive secondary metabolites from endophytic Fungi: a review of Biotechnological Production and their potential applications. In: Atta-ur-Rahman, editor. Studies in Natural products Chemistry. Elsevier; 2016. pp. 189–205.

[2] Butler MM, Williams JD, Peet NP, Moir DT, Panchal RG, Bavari S (2010). Comparative in vitro activity profiles of novel bis-indole antibacterials against gram-positive and gram-negative clinical isolates. Antimicrob Agents Chemother.

[3] Kapoore RV, Padmaperuma G, Maneein S, Vaidyanathan S (2022). Co-culturing microbial consortia: approaches for applications in biomanufacturing and bioprocessing. Crit Rev Biotechnol.

[4] Li F, Yan S, Huang Z, Gao W, Zhang S, Mo S (2021). Inducing new bioactive metabolites production from coculture of* Pestalotiopsis* sp. and *Penicillium bialowiezense*. Bioorg Chem.

[5] Ogawa M, García-Martínez T, Bisson L, Mauricio JC, Moreno J, Moreno-García J (2020). Mapping the intracellular metabolome of yeast biocapsules - spherical structures of yeast attached to fungal pellets. N Biotechnol.

[6] Oh D-C, Kauffman CA, Jensen PR, Fenical W (2007). Induced production of emericellamides a and B from the marine-derived fungus *Emericella* sp. in competing co-culture. J Nat Prod.

[7] Cueto M, Jensen PR, Kauffman C, Fenical W, Lobkovsky E, Clardy J (2001). Pestalone, a New Antibiotic produced by a Marine Fungus in response to bacterial challenge. J Nat Prod.

[8] Anteneh YS, Yang Q, Brown MH, Franco CMM (2022). Factors affecting the isolation and diversity of marine sponge-associated bacteria. Appl Microbiol Biotechnol.

[9] Gardes M, Bruns TD (1993). ITS primers with enhanced specificity for basidiomycetes - application to the identification of mycorrhizae and rusts. Mol Ecol.

[10] Tamura K, Nei M, Kumar S (2004). Prospects for inferring very large phylogenies by using the neighbor-joining method. Proc Natl Acad Sci.

[11] Sabdaningsih A, Liu Y, Mettal U, Heep J, Riyanti, Wang L (2020). A New Citrinin Derivative from the Indonesian Marine Sponge-Associated Fungus *Penicillium citrinum*. Mar Drugs.

[12] Alhadrami HA, Orfali R, Hamed AA, Ghoneim MM, Hassan HM, Hassane ASI (2021). Flavonoid-coated gold nanoparticles as efficient antibiotics against Gram-negative Bacteria—evidence from in Silico-supported in Vitro studies. Antibiotics.

[13] Elkhouly H, Hamed A, Hosainy A, Ghareeb M, Sidkey N, Sidkey N (2021). Bioactive secondary metabolite from endophytic *Aspergillus tubenginses* ASH4 isolated from *Hyoscyamus muticus*: Antimicrobial, Antibiofilm, antioxidant and anticancer activity. Pharmacognosy J.

[14] Khedr WE, Shaheen MNF, Elmahdy EM, Bendary MAE, Hamed AA, Mohamedin AH (2023). Silver and gold nanoparticles: eco-friendly synthesis, antibiofilm, antiviral, and anticancer bioactivities. Prep Biochem Biotechnol.

[15] Brand-Williams W, Cuvelier ME, Berset C (1995). Use of a free radical method to evaluate antioxidant activity. LWT - Food Science and Technology.

[16] Shaker KH, Zohair MM, Hassan AZ, Sweelam HM, Ashour WE (2022). LC–MS/MS and GC–MS based phytochemical perspectives and antimicrobial effects of endophytic fungus *Chaetomium ovatoascomatis* isolated from* Euphorbia milii*. Arch Microbiol.

[17] Agour MA, Hamed AA, Ghareeb MA, Abdel-Hamid EAA, Ibrahim MK. Bioactive secondary metabolites from marine *Actinomyces* sp. AW6 with an evaluation of ADME-related physicochemical properties. Arch Microbiol. 2022;204. 10.1007/s00203-022-03092-5 PMC9343302 35913539

[18] Daina A, Michielin O, Zoete V (2017). SwissADME: a free web tool to evaluate pharmacokinetics, drug-likeness and medicinal chemistry friendliness of small molecules. Sci Rep.

[19] Ainsworth GC, Hawksworth DL, Kirk PM, Sutton BC, Pegler DN (1971). Ainsworth & Bisby’s dictionary of the fungi.

[20] Saitou N, Nei M (1987). The neighbor-joining method: a new method for reconstructing phylogenetic trees. Mol Biol Evol.

[21] Felsenstein J (1985). Confidence limits on phylogenies: an Approach using the bootstrap. Evolution.

[22] Kumar S, Stecher G, Li M, Knyaz C, Tamura K (2018). MEGA X: Molecular Evolutionary Genetics Analysis across Computing platforms. Mol Biol Evol.

[23] Moubasher H, Elkholy A, Sherif M, Zahran M, Elnagdy S (2022). *In Vitro* Investigation of the Impact of Bacterial–Fungal Interaction on Carbapenem-resistant *Klebsiella pneumoniae*. Molecules.

[24] Nicault M, Zaiter A, Dumarcay S, Chaimbault P, Gelhaye E, Leblond P (2021). Elicitation of antimicrobial active compounds by Streptomyces-Fungus Co-cultures. Microorganisms.

[25] Muhammad MH, Idris AL, Fan X, Guo Y, Yu Y, Jin X et al. Beyond risk: bacterial biofilms and their regulating approaches. Front Microbiol. 2020;11. 10.3389/fmicb.2020.00928 PMC7253578 32508772

[26] Donlan RM, Costerton JW (2002). Biofilms: survival mechanisms of clinically relevant microorganisms. Clin Microbiol Rev.

[27] Salimi F, Almasi F, Mohammadipanah F, Abdalla MA (2022). A comparative review of plant and microbial antioxidant secondary metabolites. Appl Food Biotechnol.

[28] Wu J, Kaewnarin K, Nie X, Li Q, He N, Huang J (2021). Biological activities of a polysaccharide from the coculture of *Ganoderma lucidum* and *Flammulina velutipes* mycelia in submerged fermentation. Process Biochem.

[29] Sutthiphatkul T, Mangmool S, Rungjindamai N, Ochaikul* D. Characteristics and antioxidant activities of Kombucha from Black Tea and Roselle by a mixed starter culture. Curr Appl Sci Technol. 2023;23.

[30] Potts RO, Guy RH (1992). Predicting skin permeability. Pharm Res.

[31] Rutkowska E, Pajak K, Jóźwiak K (2013). Lipophilicity–methods of determination and its role in medicinal chemistry. Acta Pol Pharm.

[32] Banerjee P, Eckert AO, Schrey AK, Preissner R (2018). ProTox-II: a webserver for the prediction of toxicity of chemicals. Nucleic Acids Res.

