# Supplementary material for: Induction of antimicrobial, antioxidant metabolites production by co-cultivation of two red-sea-sponge-associated Aspergillus sp. CO2 and Bacillus sp. COBZ21
Source: BMC Biotechnol. 2024 Jan 17;24:3. doi: 10.1186/s12896-024-00830-z (PMC10795289; doi:10.1186/s12896-024-00830-z)
Supplement: Supplementary file 1 — Supplementary Material 1 [file 12896_2024_830_MOESM1_ESM.docx]

**Induction of Antimicrobial, Antioxidant Metabolites production by Co-Cultivation of Two Red-Sea-Sponge-Associated *Aspergillus* sp. CO2 and *Bacillus* sp. COBZ21**

Ahmed A. Hamed^1*^, Mosad A. Ghareeb^2,*^, Ayda K. Kelany^3^, Mohamed Abdelraof^1^, Hoda A. Kabary^4^, Nariman R. Soliman^5^, Mohamed E. Elawady^6^

^1^Microbial Chemistry Department, National Research Centre, Dokki, Cairo, 12622, Egypt

^2^Medicinal Chemistry Department, Theodor Bilharz Research Institute, Kornaish El-Nile, Warrak El-Hadar, Imbaba (P.O. 30), Giza 12411, Egypt https://orcid.org/0000-0002-8398-1937

^3^Department of Genomic Medicine, Cairo University, Giza, Egypt

^4^Department Agricultural Microbiology, National Research Center, 33 El Buhouth St., Dokki, 12622, Giza, Egypt

^5^Dairy Science Department, National Research Center, Dokki, Cairo, Egypt. narimanramadan24@gmail.com.

^6^Microbial Biotechnology Department, Biotechnology Research Institute National Research Centre, Cairo, Egypt. https://orcid.org/0000-0001-5155-3949

*Correspondence: Mosad A. Ghareeb; E-mail: m.ghareeb@tbri.gov.eg; Ahmed A. Hamed; E-mail: ahmedshalbio@gmail.com

**Abstract**

The growing spread of infectious diseases has become a potential global health threat to human beings. According to WHO reports, in this study, we investigated the impact of co-cultivating the isolated endophytic fungus *Aspergillus* sp. CO2 and *Bacillus* sp. COBZ21 as a method to stimulate the production of natural bioactive substances. (GC/MS)-based metabolomics profiling of two sponge-associated microbes, Aspergillus sp. CO2 and Bacillus sp. COBZ21, revealed that the co-culture of these two isolates induced the accumulation of metabolites that were not traced in their axenic cultures. By detection of different activities of extracts of Bacillus sp. COBZ21 and Aspergillus sp. CO2 and coculture between Bacillus sp. COBZ21 and Aspergillus sp. CO2. It was noted that the coculture strategy was the reason for a notable increase in some different activities, such as the antimicrobial activity, which showed potent activity against *Escherichia coli* ATCC 25922, *Staphylococcus aureus* NRRLB-767, and *Candida albicans* ATCC 10231. The antibiofilm activity showed significant biofilm inhibitory activity toward Bacillus subtilis ATCC 6633, Pseudomonas aeruginosa ATCC 10145, and Staph aureus NRRLB-767, with activity up to 53.66, *71*.17, and 47.89%, while it showed low activity against E. coli ATCC 25922, while the antioxidant activity based on the DPPH assay showed maximum activity (75.25%). GC-MS investigations revealed the presence of variable chemical constituents belonging to different chemical categories, which reflected their chemical diversity. The main components are (+-) cis-Deethylburnamine (2.66%), Bis(3,6,9,12-tetraoxapentaethylene) crowno-N,N,N',N'-tetra methylpphanediamine (2.48%), and 11-phenyl-2,4,6,8-tetra(2-thienyl)-11-aza-5,13-dithiaeteracyclo[7.3.0.1(2,8).0(3,7)] trideca-3,6-diene-10,12,13-trione (3.13%), respectively, for Bacillus sp. axenic culture, Aspergillus sp. CO2, Aspergillus sp. CO2, and Bacillus sp. COBZ21 coculture. By studying the ADME-related physicochemical properties of coculture extract, the compound showed log P_o/w_ values above 5 (8.82). The solubility of the substance was moderate. In order to provide a comprehensive definition of medicinal chemistry and leadlikness, it is important to note that the latter did not meet the criteria outlined in the rule of three (RO3). The toxicity prediction of the coculture extract was performed using the ProTox II web server, which showed that the selected compound has no pronounced toxicity.

**Keywords:** Coculture, fungi, bacteria, Antimicrobial, Antioxidant, Antibiofilm,


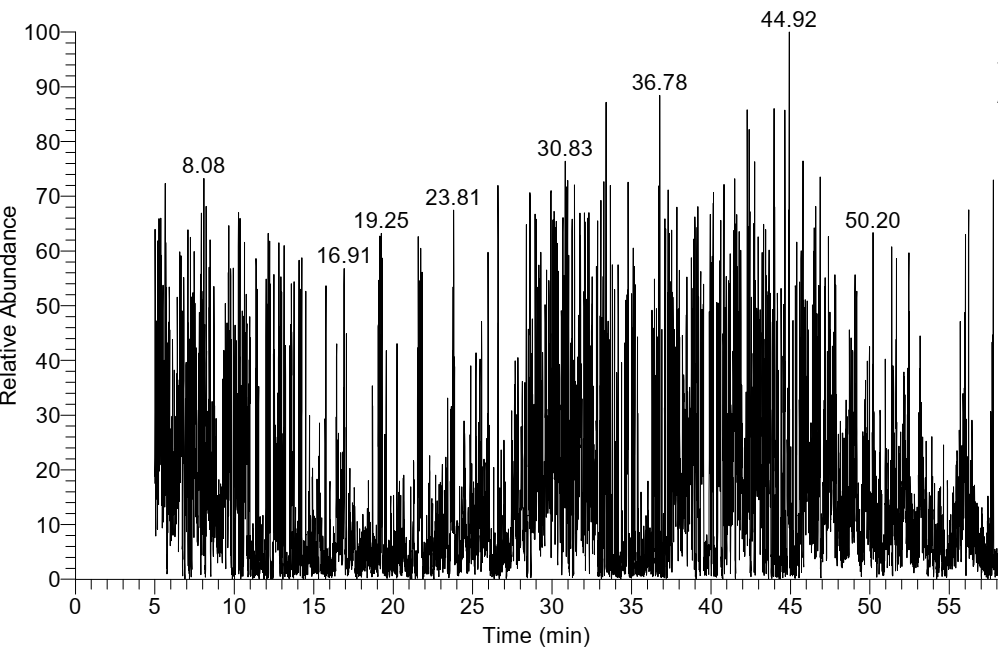


**Fig. 1S.** GC-MS chromatogram of *Bacillus* sp. COBZ21.


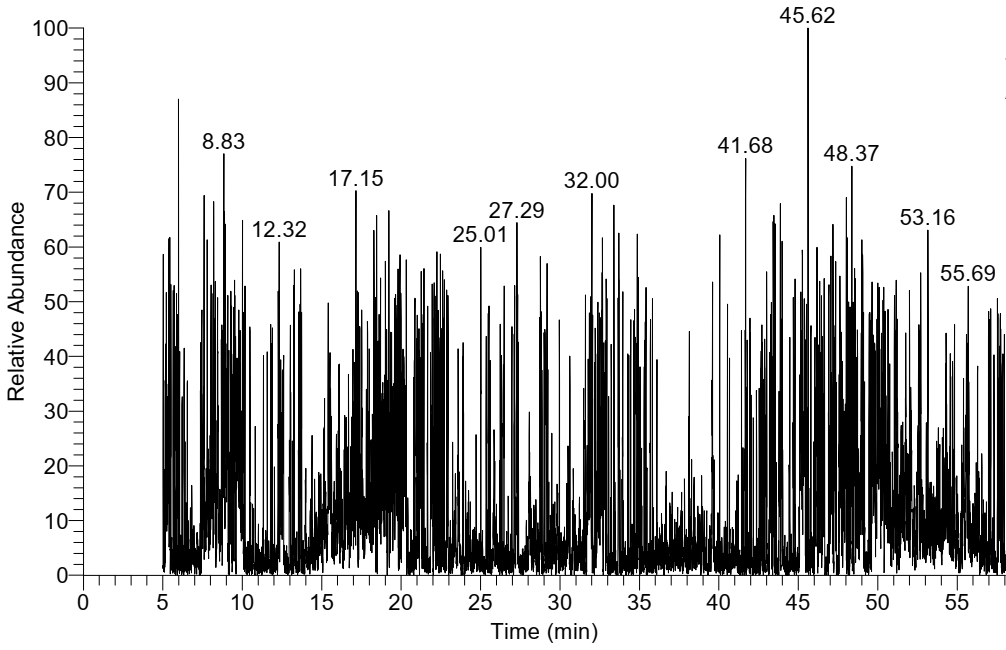


**Fig. 2S.** GC-MS chromatogram of *Aspergillus sp.* CO2.


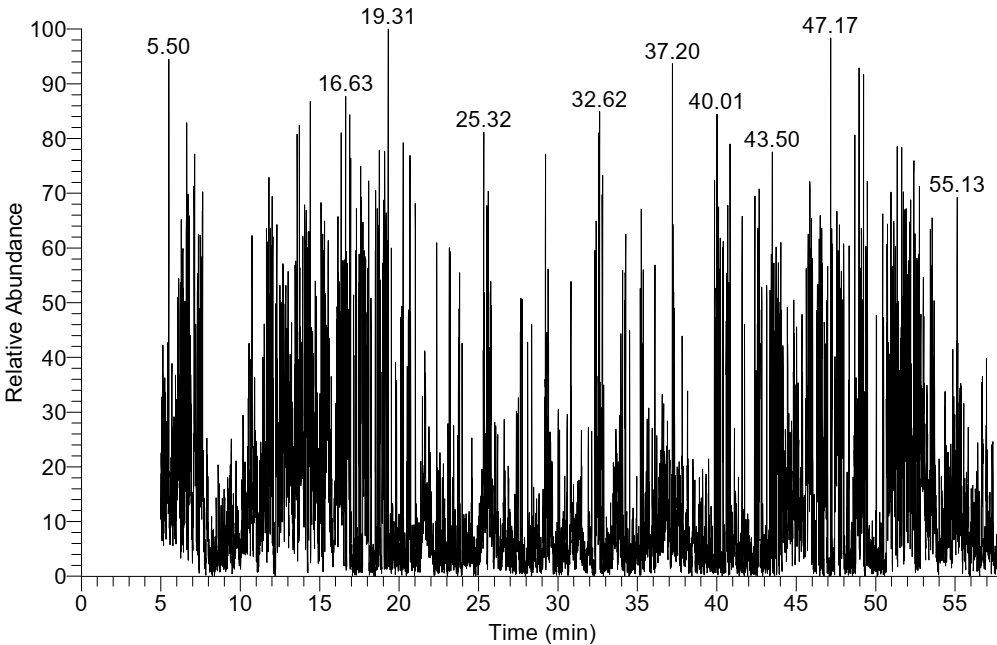


**Fig. 3S.** GC-MS chromatogram of *Aspergillus* sp. CO2 and *Bacillus* sp. COBZ21 coculturing.
